# Stellate ganglion phototherapy for electrical storm during mechanical circulatory support in acute coronary syndrome: a case series

**DOI:** 10.1093/ehjcr/ytag815

**Published:** 2026-10-30

**Authors:** Yuki Arakawa, Takehiro Hashikata, Yuki Ikeda, Nobuhiro Sato, Hidehira Fukaya, Junya Ako

**Affiliations:** Department of Cardiovascular Medicine, Kitasato University School of Medicine, 1-15-1 Kitasato, Minami-ku, Sagamihara, Kanagawa 252-0374, Japan; Department of Cardiovascular Medicine, Kitasato University School of Medicine, 1-15-1 Kitasato, Minami-ku, Sagamihara, Kanagawa 252-0374, Japan; Department of Cardiovascular Medicine, Kitasato University School of Medicine, 1-15-1 Kitasato, Minami-ku, Sagamihara, Kanagawa 252-0374, Japan; Department of Cardiovascular Medicine, Kitasato University School of Medicine, 1-15-1 Kitasato, Minami-ku, Sagamihara, Kanagawa 252-0374, Japan; Department of Cardiovascular Medicine, Kitasato University School of Medicine, 1-15-1 Kitasato, Minami-ku, Sagamihara, Kanagawa 252-0374, Japan; Department of Cardiovascular Medicine, Kitasato University School of Medicine, 1-15-1 Kitasato, Minami-ku, Sagamihara, Kanagawa 252-0374, Japan

**Keywords:** Electrical storm, Stellate ganglion phototherapy, Acute coronary syndrome, Mechanical circulatory support, Case report

## Abstract

**Background:**

Electrical storm (ES) during mechanical circulatory support (MCS) in the setting of acute coronary syndrome (ACS) represents a life-threatening condition driven by sympathetic hyperactivation and repolarization abnormalities. While stellate ganglion block is recognized as an effective autonomic modulation strategy for drug-refractory ventricular arrhythmias, its use may raise concerns regarding bleeding complications in patients receiving systemic anticoagulation. Stellate ganglion phototherapy (SGP) is a non-invasive alternative, but its clinical utility for ES under MCS remains unclear.

**Case summary:**

This case series describes two patients with drug-refractory ES complicating ACS during MCS management. Patient 1, a 78-year-old woman with cardiogenic shock, developed recurrent ventricular fibrillation (VF) with QT prolongation while on veno-arterial extracorporeal membrane oxygenation. Overdrive pacing caused haemodynamic deterioration, and conventional antiarrhythmic drugs failed. Following SGP initiation, no further VF requiring electrical shocks occurred. Extracorporeal membrane oxygenation was subsequently withdrawn. Patient 2, a 77-year-old man, developed recurrent torsades de pointes/VF with QT prolongation after left main revascularization under Impella support. Following SGP initiation, no further episodes requiring electrical shocks occurred. Impella was subsequently removed.

**Discussion:**

Stellate ganglion phototherapy may represent a non-invasive autonomic modulation strategy for drug-refractory ES in ACS patients requiring MCS. Although concomitant therapies and gradual recovery from myocardial ischaemia may have contributed to arrhythmia suppression, the temporal association suggests a potential contribution of SGP. Stellate ganglion phototherapy may serve as a bridge to recovery when invasive approaches pose additional challenges.

Learning points

- To recognize sympathetic hyperactivation and repolarization abnormalities as important contributors to electrical storm (ES) complicating acute coronary syndrome
- To recognize stellate ganglion phototherapy (SGP) as a non-invasive neuromodulation option for ES during mechanical circulatory support (MCS), particularly when bleeding concerns limit stellate ganglion blockade
- To recognize the potential role of SGP as a bridge to recovery through arrhythmia stabilization during MCS

## Introduction

Electrical storm (ES) is a life-threatening cluster of ventricular arrhythmias that can complicate acute coronary syndrome (ACS) or advanced heart failure, often hindering weaning from mechanical circulatory support (MCS) in critically ill patients.^1^ In ACS, acute sympathetic hyperactivation and associated repolarization abnormalities are recognized contributors to malignant ventricular arrhythmias, whereas arrhythmic activity may subside once the acute phase resolves.^2,3^ Stellate ganglion block (SGB) has been reported to effectively suppress drug-refractory ES; however, its invasive nature may raise concerns regarding bleeding complications in patients receiving systemic anticoagulation. Stellate ganglion phototherapy (SGP), a non-invasive alternative to SGB, employs low-level light irradiation to modulate sympathetic activity and has demonstrated sympatholytic effects in both experimental and clinical settings.^4,5^ Unlike chronic structural heart diseases where ES is often driven by irreversible fixed substrates, ES in ACS is primarily triggered by a transient sympathetic surge, suggesting that SGP could serve a unique role as a temporary bridge to recovery. Nevertheless, its therapeutic potential for controlling life-threatening arrhythmias in ACS patients requiring MCS remains unclear. Here, we describe two cases of drug-refractory ES secondary to ACS during MCS management in which ES subsided following SGP initiation.

## Summary figure

**Figure:**
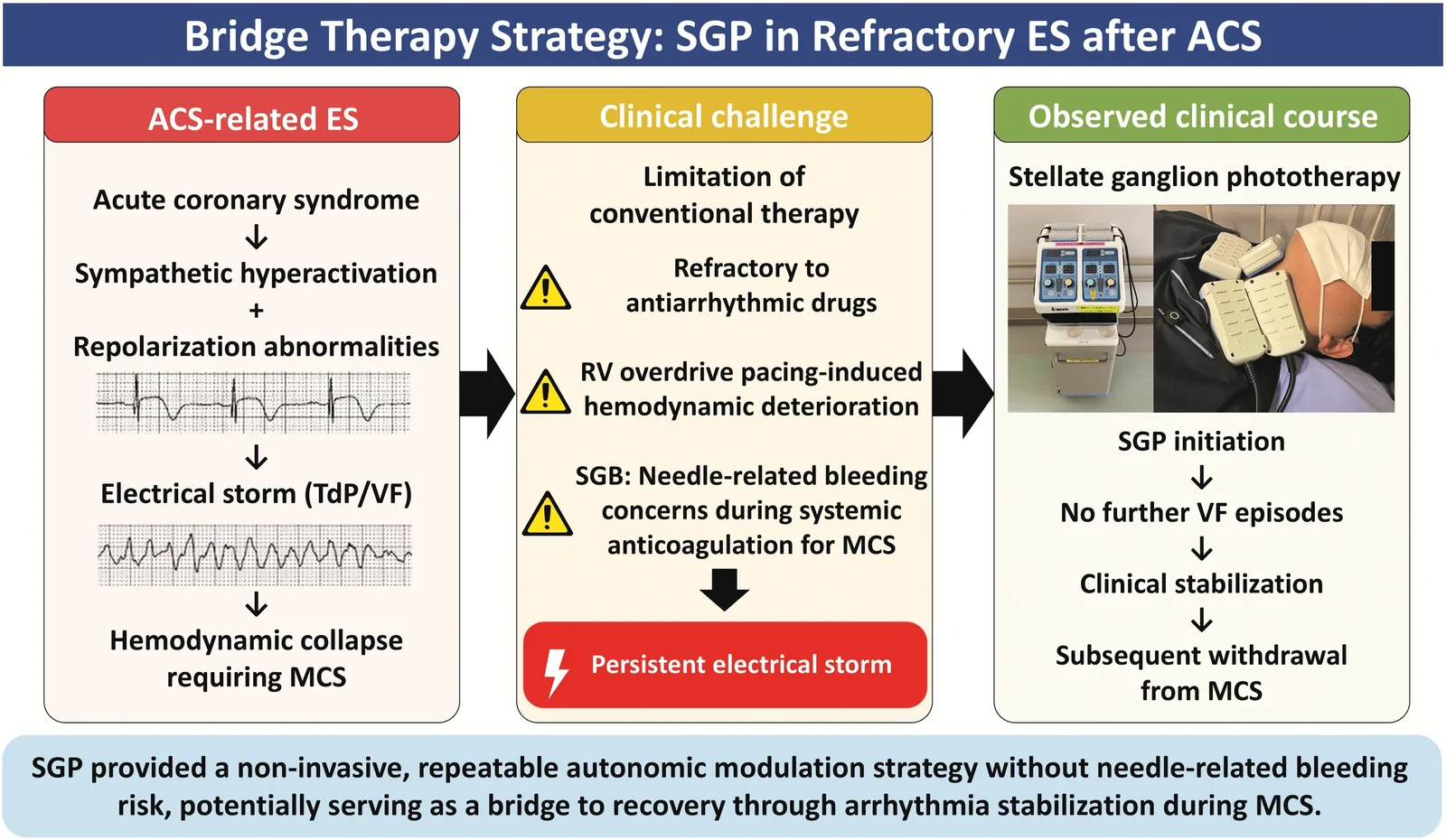


## Case presentation

### Patient 1

A 78-year-old woman with a history of diabetes mellitus was admitted with progressive dyspnoea and was diagnosed with acute decompensated heart failure (ADHF). On admission, her heart rate was 120 beats/min, blood pressure was 194/130 mmHg, and oxygen saturation was 77% on room air, with bilateral coarse crackles on auscultation. Transthoracic echocardiography revealed a left ventricular ejection fraction (LVEF) of 27% with hypokinesis in the anterior wall, suggesting ischaemic cardiomyopathy (ICM). As there were no chest pain episodes or elevation of cardiac enzymes, initial management focused on heart failure treatment. On hospital Day 5, she developed ventricular fibrillation (VF). Emergency coronary angiography (CAG) demonstrated critical stenosis of the left main trunk and left anterior descending artery (LAD), and percutaneous coronary intervention (PCI) was performed with implantation of drug-eluting stents (DES) in the left main trunk (3.25 × 15 mm) and mid-LAD (2.5 × 23 mm), followed by drug-coated balloon angioplasty in the distal LAD (2.0 × 30 mm), achieving successful revascularization (*Figure 1A–D*). However, VF recurred on Day 6, progressing to an ES. Veno-arterial extracorporeal membrane oxygenation (VA-ECMO) and an intra-aortic balloon pump (IABP) were initiated for haemodynamic support. Repeat CAG revealed stent thrombosis in the mid-LAD and proximal stent-edge dissection. Percutaneous coronary intervention was performed with implantation of a DES (3.0 × 15 mm), achieving complete revascularization (*Figure 1E* and *F*). An electrocardiogram showed T-wave inversion in the anterior leads and QT prolongation (QTc 524 ms) (*Figure 2A*). Despite optimal revascularization and antiarrhythmic drug therapy (*Figure 4*), drug-refractory ES persisted, characterized by recurrent episodes of torsades de pointes (TdP) degenerating into VF (*Figure 2B*). Right ventricular apical overdrive pacing with a temporary pacemaker suppressed VF but led to haemodynamic deterioration; arrhythmias recurred immediately after pacing cessation, precluding ECMO weaning. Given concerns about bleeding associated with SGB during systemic anticoagulation, SGP was initiated on Day 14. During the 3 days preceding SGP initiation (Days 11–13), three electrical shocks were required for VF, including two on Day 13 (*Figure 4*). Stellate ganglion phototherapy was performed using a xenon light irradiation system (EXCEL Glanz X2; Kokusai Commerce Inc., Tokyo, Japan). Bilateral cervical irradiation over the anatomical region of the stellate ganglion was applied for 10 min daily during the acute phase (*Figure 3*). The last VF episode occurred before the first SGP session, with no recurrence of such episodes thereafter. Veno-arterial extracorporeal membrane oxygenation and IABP were subsequently withdrawn on Days 19 and 21, respectively. Stellate ganglion phototherapy was administered daily from Day 14 to Day 33, comprising 20 sessions. As this was our first experience with SGP for VF, no predefined stopping criteria were applied; treatment was discontinued after sustained absence of VF episodes and clinical stabilization. The QTc interval on Day 21 was 432 ms, indicating that the previously observed QT prolongation had resolved (*Figure 2C*). The patient was discharged on Day 82. The clinical timeline, including the timing and dosages of antiarrhythmic medications, is shown in *Figure 4*.

**Figure 1:**
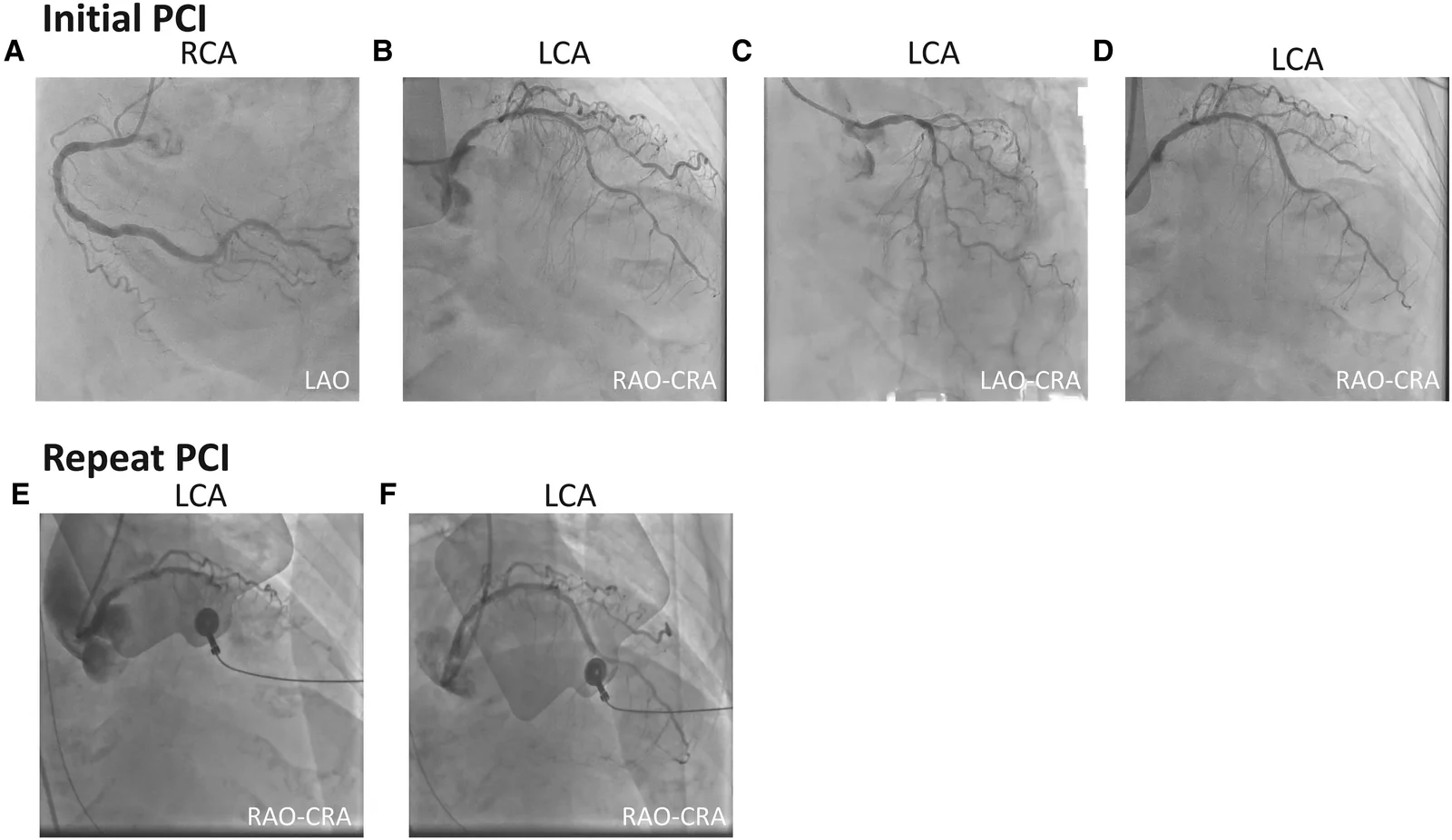
Coronary angiographic findings of Case 1. (*A–C*) Initial coronary angiography (CAG) demonstrating severe stenosis of the left main trunk and proximal left anterior descending artery (LAD). (*D*) Final angiographic result after the initial percutaneous coronary intervention (PCI). (*E*) Repeat CAG showing stent thrombosis in the LAD. (*F*) Final angiographic result after repeat PCI. Abbreviations: PCI, percutaneous coronary intervention; RCA, right coronary artery; LCA, left coronary artery; LAO, left anterior oblique; RAO, right anterior oblique; CRA, cranial.

**Figure 2:**
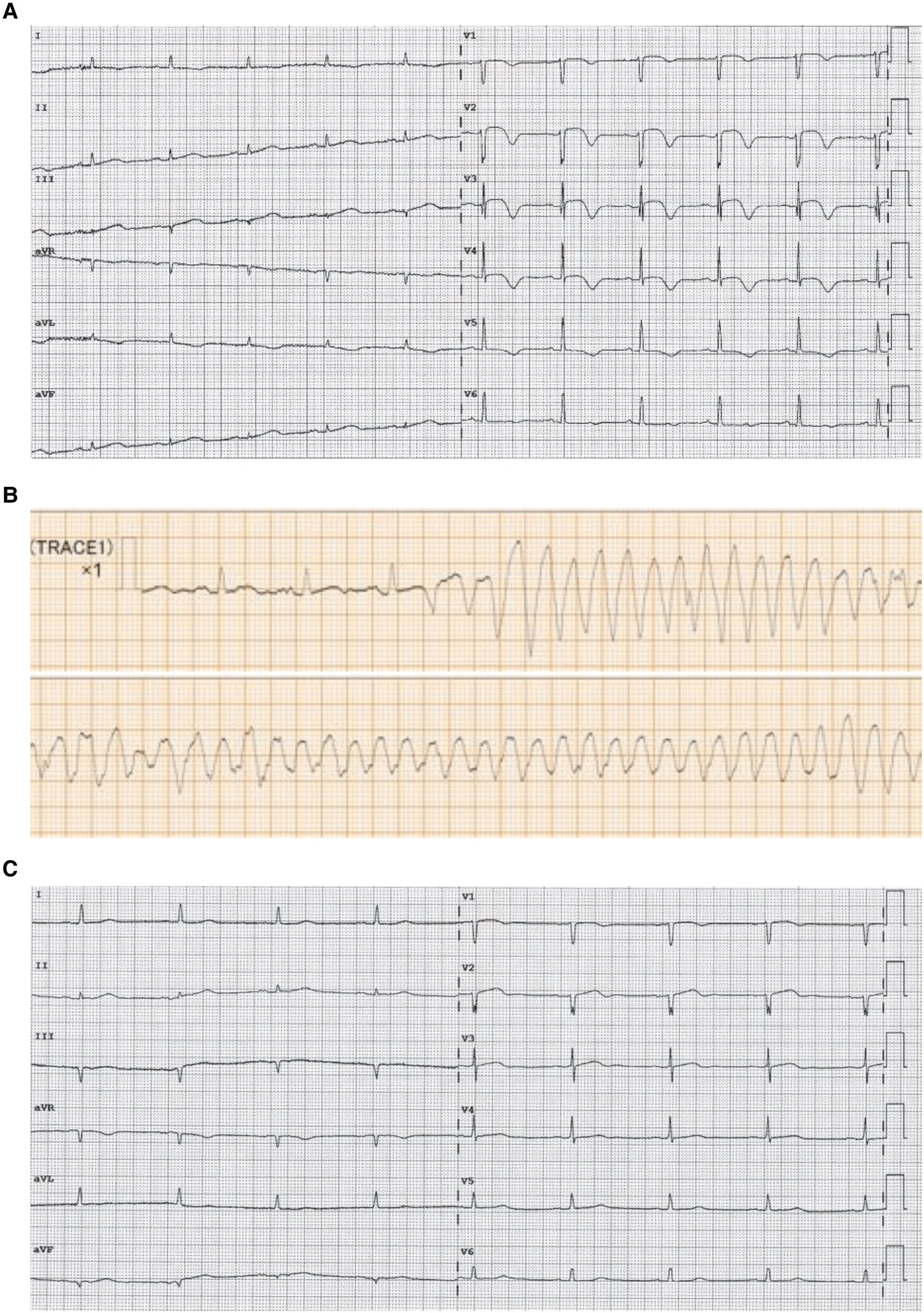
Electrocardiographic findings of Case 1. (*A*) Electrocardiogram obtained after the second percutaneous coronary intervention on Day 6, demonstrating marked QT prolongation (QTc 524 ms). (*B*) Electrocardiographic recording on Day 6 showing torsades de pointes. (*C*) Electrocardiogram on Day 21 showing resolution of QT prolongation, with QTc shortening to 432 ms.

**Figure 3:**
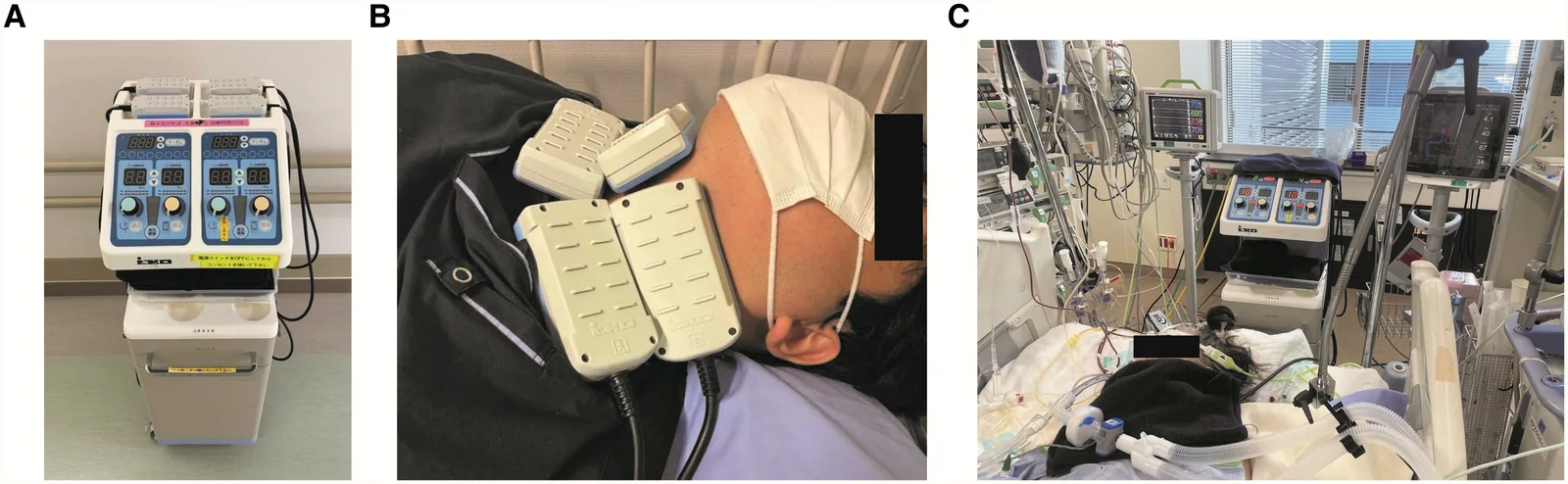
Stellate ganglion phototherapy (SGP). (*A*) Xenon light irradiation system used for SGP. (*B*) Bilateral cervical irradiation during SGP, with probes positioned over the anatomical region of the stellate ganglion (representative image). (*C*) SGP administered in a patient receiving mechanical circulatory support in the intensive care unit.

**Figure 4:**
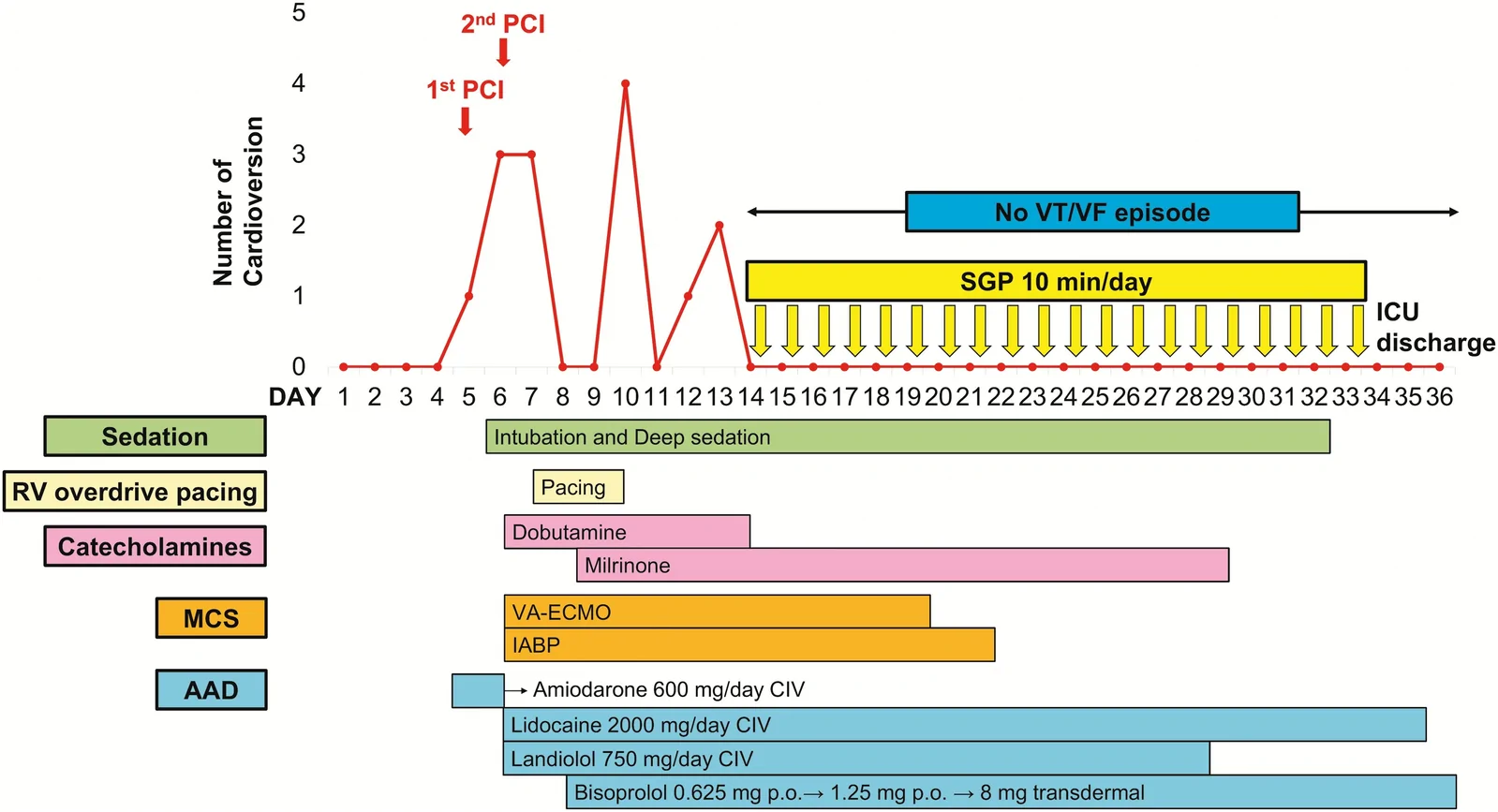
Clinical timeline of Case 1. Following ventricular fibrillation (VF), coronary revascularization was performed; however, VF storm subsequently developed, necessitating veno-arterial extracorporeal membrane oxygenation. Despite intensive antiarrhythmic therapy and deep sedation, the electrical storm persisted. Following initiation of stellate ganglion phototherapy, no further ventricular fibrillation episodes occurred. Mechanical circulatory support was subsequently withdrawn. Abbreviations: PCI, percutaneous coronary intervention; VT/VF, ventricular tachycardia/ventricular fibrillation; SGP, stellate ganglion phototherapy; ICU, intensive care unit; MCS, mechanical circulatory support; IABP, intra-aortic balloon pump; AAD, antiarrhythmic drug; CIV, continuous intravenous infusion.

### Patient 2

A 77-year-old man with a history of ICM after multivessel PCI was admitted with acute dyspnoea. On admission, his heart rate was 95 beats/min, blood pressure was 146/95 mmHg, and oxygen saturation was 95% while receiving oxygen at 4 L/min, with bilateral coarse crackles on auscultation. Transthoracic echocardiography revealed a severely reduced LVEF of 14%, and he was diagnosed with ADHF. An electrocardiogram showed abnormal Q waves in the anterior leads and QT prolongation (QTc 511 ms) (*Figure 5A*). On hospital Day 2, he developed VF following TdP (*Figure 5B*), prompting emergency CAG, which revealed a 90% stenosis of the left main trunk (LMT). An Impella CP (Cadiac Power; Abiomed, Danvers, MA, USA) was inserted for circulatory support, followed by PCI of the LMT with implantation of a DES (3.5 × 20 mm), achieving successful revascularization (*Figure 6*). Despite intensive antiarrhythmic drug therapy (*Figure 7*), VF recurred on Day 7, progressing to an ES that precluded weaning from MCS, with further QT prolongation (QTc 565 ms) (*Figure 5C*). Between episodes of ventricular arrhythmia, the heart rate remained approximately 80–100 beats/min. As the episodes did not appear to be bradycardia-dependent, overdrive pacing was not attempted. Despite QT prolongation, amiodarone was initiated on Day 9 because recurrent paroxysmal atrial fibrillation caused significant haemodynamic deterioration, necessitating rhythm control. With persistent QT prolongation and the continued need for amiodarone therapy, SGP was initiated on Day 10 for persistent ES. During the 3 days preceding SGP initiation (Days 7–9), four electrical shocks were required for VF, including one on Day 9 (*Figure 7*). The last VF episode occurred before the first SGP session, with no recurrence of such episodes thereafter. Impella CP was successfully removed on Day 15. Stellate ganglion phototherapy was administered daily from Day 10 to Day 23, comprising 14 sessions. In consultation with the anaesthesiology team, a 14-day course was adopted at our institution to cover the acute phase, with continued assessment of arrhythmia burden and clinical status. Treatment was discontinued upon completion of the course, given the sustained absence of recurrent VF episodes. The QTc interval on Day 15 was 415 ms, indicating that the previously observed QT prolongation had resolved (*Figure 5D*). However, the patient ultimately died of sepsis on Day 46. The clinical timeline, including the timing and dosages of antiarrhythmic medications, is shown in *Figure 7*.

**Figure 5:**
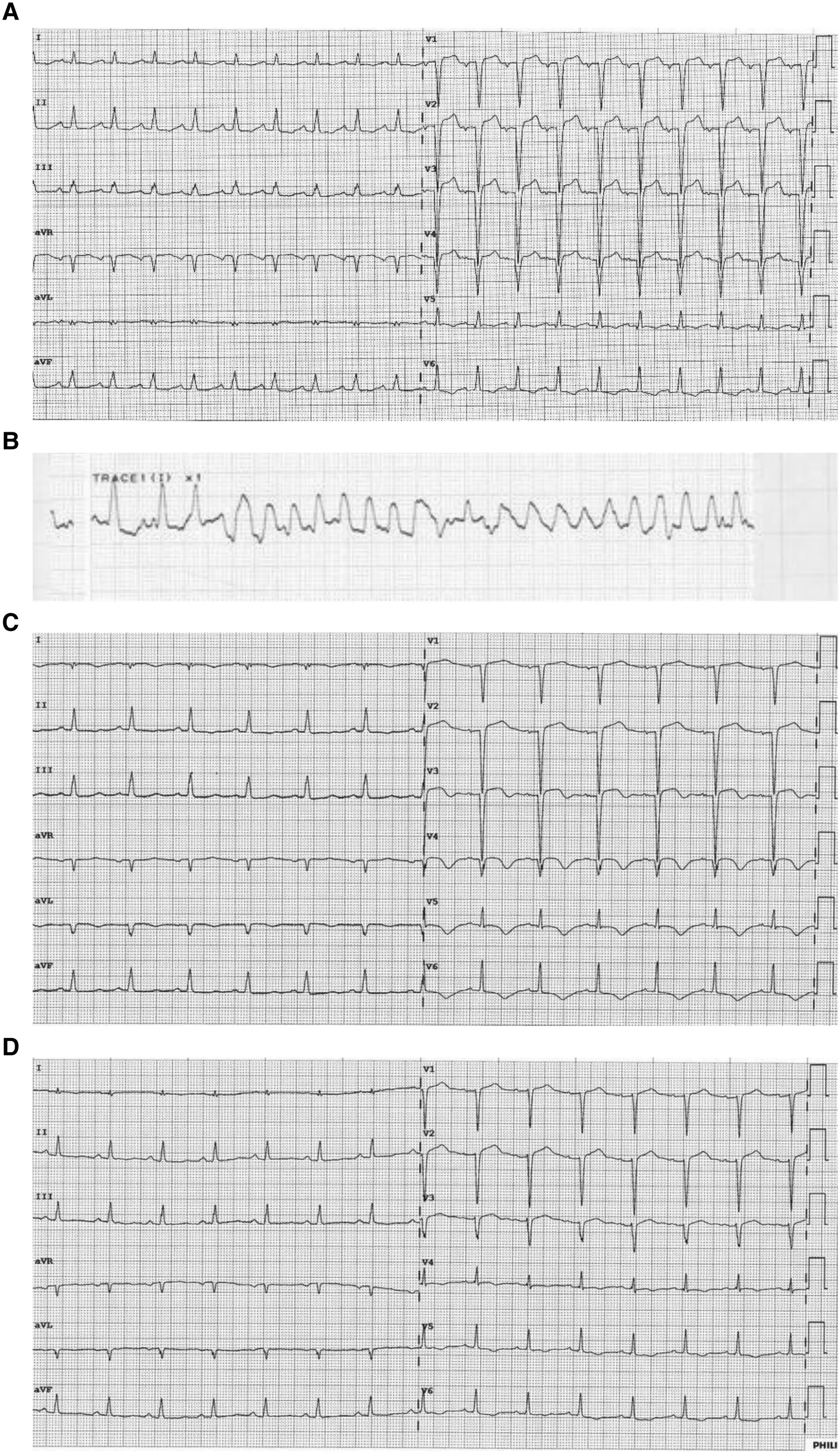
Electrocardiographic findings in Case 2. (*A*) Electrocardiogram obtained on admission, demonstrating marked QT prolongation (QTc 511 ms). (*B*) Electrocardiogram on Day 2 showing ventricular fibrillation following torsades de pointes. (*C*) Electrocardiogram on Day 7 demonstrating further QT prolongation (QTc 565 ms). (*D*) Electrocardiogram on Day 15 showing resolution of QT prolongation, with QTc shortening to 415 ms.

**Figure 6:**
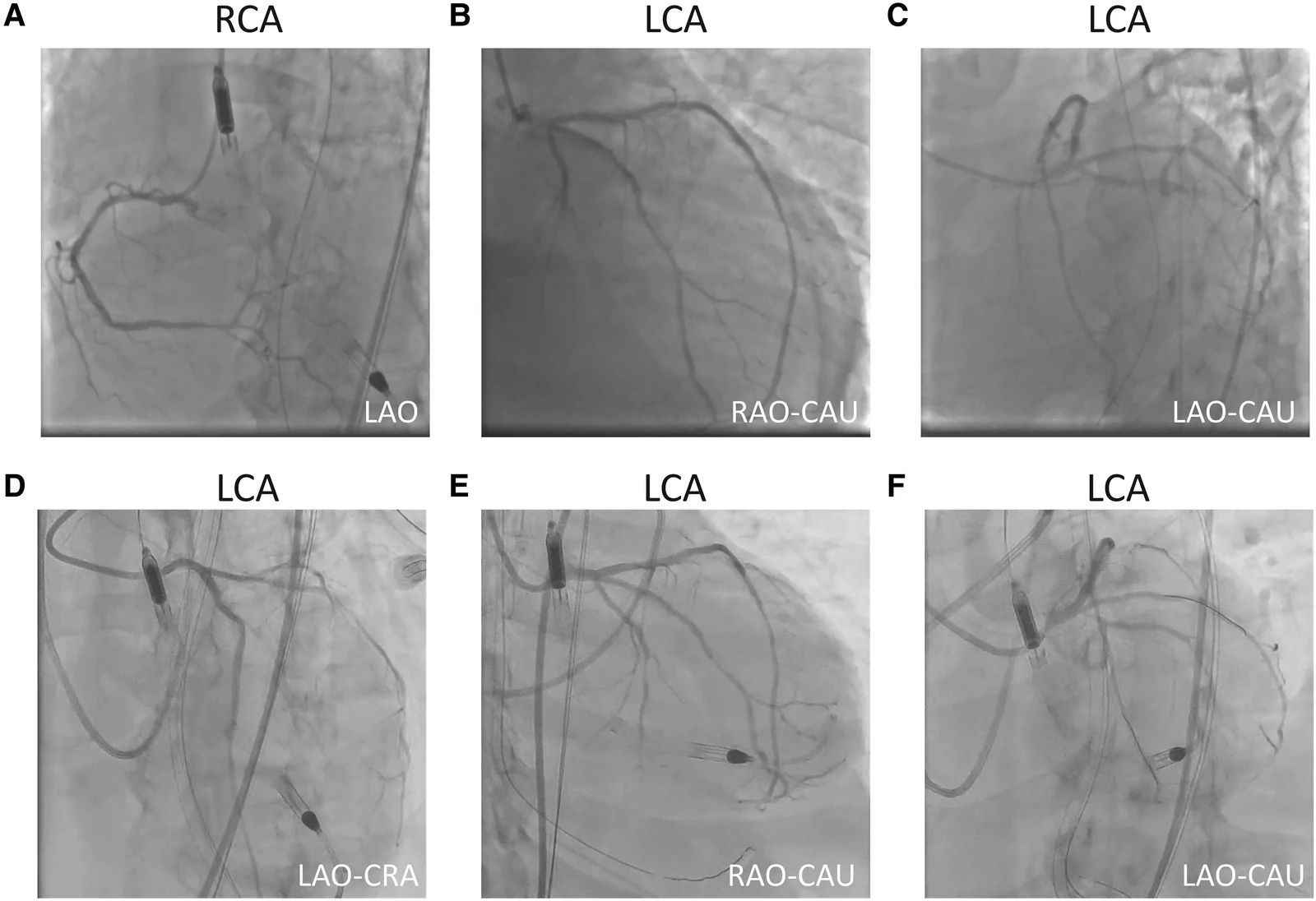
Coronary angiographic findings of Case 2. (*A–C*) Coronary angiography demonstrating severe stenosis of the left main trunk. (*D–F*) Impella CP placement for circulatory support and final angiographic images after stent implantation, demonstrating successful revascularization. Abbreviations: RCA, right coronary artery; LCA, left coronary artery; LAO, left anterior oblique; RAO, right anterior oblique; CAU, caudal; CRA, cranial.

**Figure 7:**
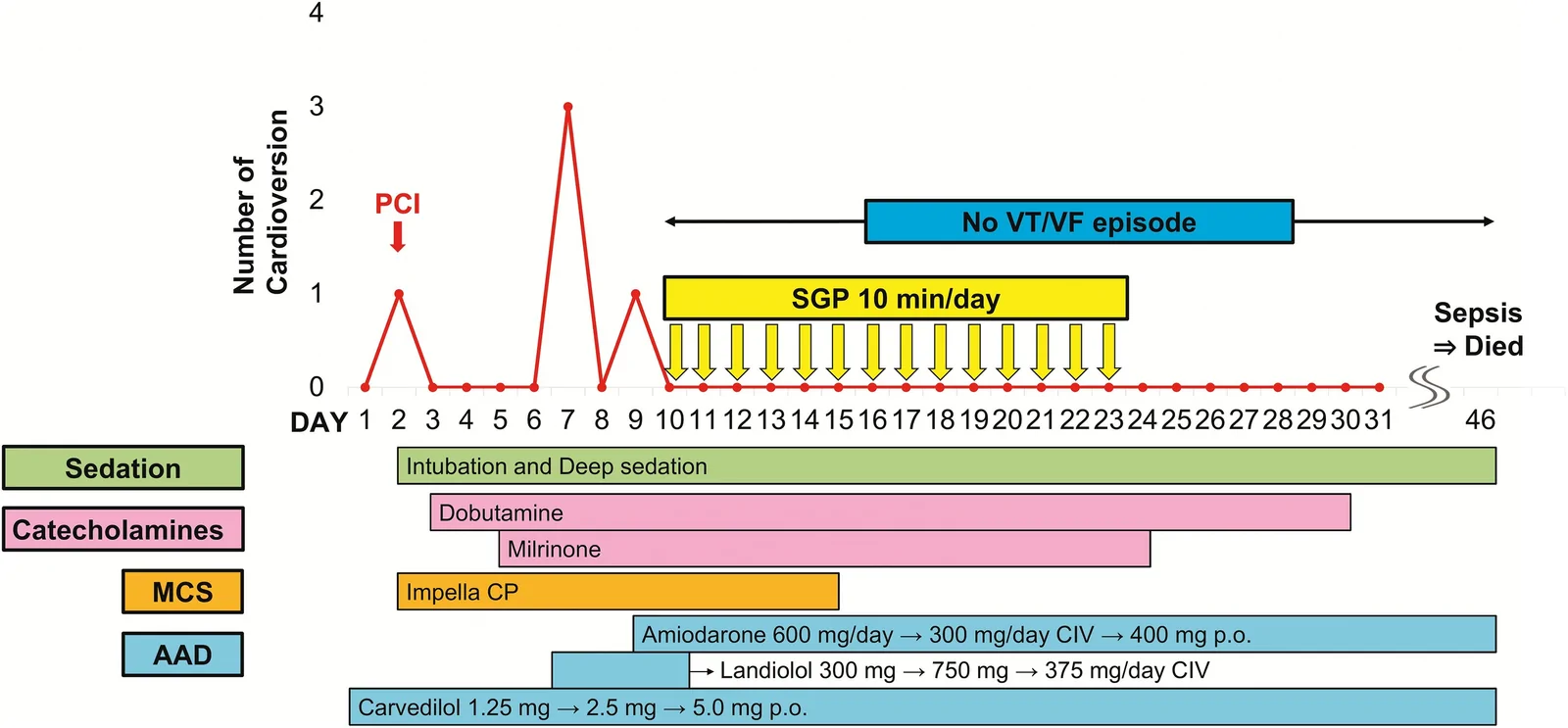
Clinical timeline of Case 2. Following ventricular fibrillation (VF), coronary revascularization was performed; however, VF storm persisted despite intensive antiarrhythmic therapy and deep sedation, precluding weaning from mechanical circulatory support (MCS). Following stellate ganglion phototherapy, no further VF episodes occurred. MCS was subsequently withdrawn. Abbreviations: PCI, percutaneous coronary intervention; VT/VF, ventricular tachycardia/ventricular fibrillation; SGP, stellate ganglion phototherapy; MCS, mechanical circulatory support; AAD, antiarrhythmic drug.

## Discussion

This case series highlights the potential utility of SGP as a non-invasive neuromodulation strategy for drug-refractory ES during the acute phase of ACS in patients receiving MCS and systemic anticoagulation. Electrical storm occurring during ACS is associated with particularly poor prognosis and remains a major therapeutic challenge despite contemporary revascularization strategies.^6^ Current European Society of Cardiology (ESC) guidelines and expert consensus recognize autonomic modulation, including SGB, and catheter ablation as therapeutic options for drug-refractory ES.^7,8^ Although percutaneous SGB has been performed in critically ill patients requiring MCS,^9^ systemic anticoagulation may raise concerns regarding needle-related bleeding. Catheter ablation is another important option and, in patients with refractory VF storm after myocardial infarction, may target Purkinje-related triggers.^10^ Given the severe haemodynamic instability requiring MCS during the acute phase of ACS, we prioritized SGP for acute arrhythmia control because it is non-invasive and readily applicable at the bedside. Catheter ablation was considered as a subsequent option if ES persisted. The fundamental mechanism driving ES in the present cases was likely a vicious cycle of sympathetic hyperactivation and repolarization abnormalities. Both patients exhibited marked QT prolongation and TdP progressing to VF. Acute myocardial ischaemia, compounded by severe heart failure, can promote sympathetic activation. This sympathetic overactivity may exacerbate repolarization abnormalities, contributing to QT prolongation and creating a vulnerable arrhythmogenic substrate.^2,11^ Furthermore, conventional therapies often reach their limits in such haemodynamic collapse. In Patient 1, right ventricular apical overdrive pacing could not be continued because of significant haemodynamic deterioration. Although not attempted in this case, atrial overdrive pacing could have been considered to increase the heart rate while preserving physiological ventricular activation, given the preserved atrioventricular conduction. Conduction system overdrive pacing with an externalized pacemaker could also have been considered as an alternative to right ventricular apical pacing. Antiarrhythmic drug therapy also failed to control the VF storm and carried potential proarrhythmic risks. Although amiodarone is an established treatment for ventricular arrhythmias, its QT-prolonging effect warrants caution in patients with marked QT prolongation and TdP.^8^ Accordingly, amiodarone was discontinued in Patient 1 after the development of TdP with marked QT prolongation. In Patient 2, however, amiodarone was continued because recurrent atrial fibrillation caused significant haemodynamic deterioration, highlighting the need to balance its potential proarrhythmic risk against the clinical need for rhythm control. Stellate ganglion phototherapy may modulate this sympathetic outflow without depressing myocardial contractility.^4,12^ Ventricular arrhythmia burden may serve as a practical measure of therapeutic response to SGP. Although reductions in serum adrenaline and the ratio of low-frequency to high-frequency power of heart rate variability (LF/HF) have been reported in healthy volunteers, their utility for response monitoring in critically ill patients remains uncertain.^5^ No standardized response-guided protocol has been established; in our patients, bilateral SGP was performed using a fixed protocol of 10 min daily while ventricular arrhythmia burden and haemodynamics were monitored. Furthermore, the clinical course of these patients highlights the potential role of SGP as a ‘bridge therapy’ in ACS. Previous reports of SGP have largely involved patients with chronic structural heart diseases in which persistent, fixed arrhythmogenic substrates predominate.^5,13,14^ In such settings, SGP often serves as an adjunctive or palliative measure, and its efficacy can be limited by the underlying irreversible scar. In contrast, ES in ACS is primarily driven by transient factors, including acute ischaemia and profound autonomic activation.^1,3^ Notably, despite complete revascularization and ongoing medical therapy, the ES persisted for several days and continued to preclude liberation from MCS in both patients. Although concomitant therapies and gradual recovery from myocardial ischaemia may have contributed to arrhythmia suppression, VF occurred in both patients on the day before SGP initiation, with no recurrence after the first SGP session. While a causal relationship cannot be definitively established in a case series and further prospective investigation is required, SGP represents a repeatable and non-invasive modality that may serve as a bridge to stabilize malignant arrhythmias during MCS management in the acute phase of ACS. Regarding safety, no adverse events were reported in healthy volunteers or 11 patients with ES, with no significant acute changes in blood pressure or electrocardiographic parameters in healthy volunteers.^5^ However, clinical experience with SGP remains limited, and specific contraindications have not been clearly defined. No SGP-related adverse events were observed in our patients. To our knowledge, this case series is among the first to specifically describe the use of SGP as a potential non-invasive bridge therapy for arrhythmia stabilization in patients with ACS-related ES receiving MCS and systemic anticoagulation.

## Lead author biography


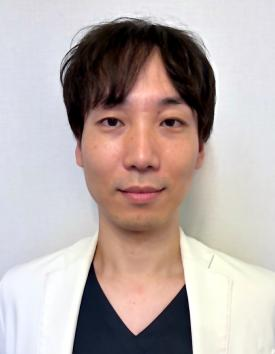


Yuki Arakawa, MD, PhD, is an assistant professor in the Department of Cardiovascular Medicine at Kitasato University School of Medicine, Japan. He is a cardiologist specializing in cardiac electrophysiology, with particular interests in catheter ablation, cardiac implantable electronic devices, and the management of complex ventricular arrhythmias.

## Data Availability

All data underlying this article are available in the article.

## References

[1] Nademanee K, Taylor R, Bailey WE, Rieders DE, Kosar EM. Treating electrical storm: Sympathetic blockade versus advanced cardiac life support-guided therapy. Circulation 2000;102:742–747. 10.1161/01.cir.102.7.742 10942741

[2] Grune J, Lewis AJM, Yamazoe M, Hulsmans M, Rohde D, Xiao L, et al Neutrophils incite and macrophages avert electrical storm after myocardial infarction. Nat Cardiovasc Res 2022;1:649–664. 10.1038/s44161-022-00094-w PMC9410341 36034743

[3] Proietti R, Sagone A. Electrical storm: incidence, prognosis and therapy. Indian Pacing Electrophysiol J 2011;11:34–42. PMC3063613 21468247

[4] Fudim M, Boortz-Marx R, Ganesh A, Waldron NH, Qadri YJ, Patel CB, et al Stellate ganglion blockade for the treatment of refractory ventricular arrhythmias: a systematic review and meta-analysis. J Cardiovasc Electrophysiol 2017;28:1460–1467. 10.1111/jce.13324 28833780

[5] Nonoguchi NM, Adachi M, Nogami A, Komatsu Y, Sato T, Ueda A, et al Stellate ganglion phototherapy using low-level laser: a novel rescue therapy for patients with refractory ventricular arrhythmias. JACC Clin Electrophysiol 2021;7:1297–1308. 10.1016/j.jacep.2021.04.015 34217659

[6] Salaun A, Martins R, Ninni S, Mohammed R, Sava R, Decaudin D, et al Characteristics and prognosis of electrical storm during acute myocardial infarction. Europace 2025;27:euaf077. 10.1093/europace/euaf077 PMC12076010 40173422

[7] Zeppenfeld K, Tfelt-Hansen J, de Riva M, Winkel BG, Behr ER, Blom NA, et al 2022 ESC guidelines for the management of patients with ventricular arrhythmias and the prevention of sudden cardiac death. Eur Heart J 2022;43:3997–4126. 10.1093/eurheartj/ehac262 36017572

[8] Lenarczyk R, Zeppenfeld K, Tfelt-Hansen J, Heinzel FR, Deneke T, Ene E, et al Management of patients with an electrical storm or clustered ventricular arrhythmias: a clinical consensus statement of the European Heart Rhythm Association of the ESC—endorsed by the Asia-Pacific Heart Rhythm Society, Heart Rhythm Society, and Latin-American Heart Rhythm Society. Europace 2024;26:euae049. 10.1093/europace/euae049 PMC10999775 38584423

[9] Motazedian P, Quinn N, Wells GA, Beauregard N, Lam E, Mathieu ME, et al Efficacy of stellate ganglion block in treatment of electrical storm: a systematic review and meta-analysis. Sci Rep 2024;14:24719. 10.1038/s41598-024-76663-9 PMC11494072 39433834

[10] Komatsu Y, Hocini M, Nogami A, Maury P, Peichl P, Iwasaki YK, et al Catheter ablation of refractory ventricular fibrillation storm after myocardial infarction: a multicenter study. Circulation 2019;139:2315–2325. 10.1161/CIRCULATIONAHA.118.037997 30929474

[11] Zipes DP . Influence of myocardial ischemia and infarction on autonomic innervation of heart. Circulation 1990;82:1095–1105. 10.1161/01.cir.82.4.1095 2205413

[12] Liao C-D, Tsauo J-Y, Chen H-C, Liou T-H. Efficacy of stellate ganglion blockade applied with light irradiation: a systemic review and meta-analysis. Am J Phys Med Rehabil 2017;96:e97–e110. 10.1097/PHM.0000000000000675 28118275

[13] Sato T, Kamada R, Koizumi T, Takenaka S, Tada A, Tsujinaga S, et al Refractory ventricular tachycardia in a patient with a left ventricular assist device successfully treated with stellate ganglion phototherapy. Can J Cardiol 2020;36:1977.e1–1977.e3. 10.1016/j.cjca.2020.08.002 32798700

[14] Takahashi K, Egami Y, Nishino M, Tanouchi J. Clinical impact of stellate ganglion phototherapy on ventricular tachycardia storm requiring mechanical circulatory support devices: a case report. Eur Heart J Case Rep 2024;8:ytae177. 10.1093/ehjcr/ytae177 PMC11060104 38690554

